# Combined expression of caveolin-1 and an activated AKT/mTOR pathway predicts reduced disease-free survival in clinically confined renal cell carcinoma

**DOI:** 10.1038/sj.bjc.6604243

**Published:** 2008-02-19

**Authors:** L Campbell, B Jasani, K Edwards, M Gumbleton, D F R Griffiths

**Affiliations:** 1 Experimental Cancer Therapeutics, School Of Pharmacy, Cardiff University, Cardiff CF10 3XF, UK; 2 Department of Pathology, School of Medicine, Cardiff University, Cardiff CF14 4XN, UK

**Keywords:** renal cell carcinoma, caveolin-1, AKT/mTOR pathway, vascular invasion, metastasis, rapamycin, prognosis

## Abstract

We previously reported that tumour-associated caveolin-1 is a potential biomarker in renal cell carcinoma (RCC), whose overexpression predicts metastasis following surgical resection for clinically confined disease. Much attention has recently focused on the AKT/mTOR pathway in a number of malignancies, including RCC. Since caveolin-1 and the AKT/mTOR signalling cascade are independently shown to be important regulators of tumour angiogenesis, we hypothesised that caveolin-1 interacts with the AKT/mTOR pathway to drive disease progression and metastasis in RCC. The aims of this study were to determine (i) the expression status of the activated AKT/mTOR pathway components (phosphorylated forms) in RCC and (ii) their prognostic value when combined with caveolin-1. Immunohistochemistry for caveolin-1, pAKT, pmTOR, pS6 and p4E-BP1 was performed on tissue microarrays from 174 clinically confined RCCs. Significantly decreased mean disease-free survival was observed when caveolin-1 was coexpressed with either pAKT (2.95 *vs* 6.14 years), pmTOR (3.17 *vs* 6.28 years), pS6 (1.45 *vs* 6.62 years) or p4E-BP1 (2.07 *vs* 6.09 years) than when neither or any one single biomarker was expressed alone. On multivariate analysis, the covariate of ‘caveolin-1/AKT’ (neither alone were influential covariates) was a significant influential indicator of poor disease-free survival with a hazard ratio of 2.13 (95% CI: 1.15–3.92), higher than that for vascular invasion. Tumours that coexpressed caveolin-1 and activated mTOR components were more likely to be larger, higher grade and to show vascular invasion. Our results provide the first clinical evidence that caveolin-1 cooperates with an activated AKT/mTOR pathway in cancer and may play an important role in disease progression. We conclude that evaluation of the ‘caveolin-1/AKT/mTOR axis’ in primary kidney tumours will identify subsets of RCC patients who require greater postoperative surveillance and more intensive treatment.

During the last 20 years, the incidence of renal cell carcinoma (RCC) in the developed world has progressively increased accounting for approximately 3% of cancers and 2% of all cancer-related deaths (Ljungberg *et al*, 2007). Renal cell carcinoma represents a heterogeneous group of neoplasms of varying clinical course even in patients with seemingly similar pathology. Two-thirds of all RCC patients have clinically localised disease at initial presentation and will undergo full surgical resection of the primary tumour. Despite this intervention, up to 40% will later develop distant metastasis for which patient outcomes are poor (Ljungberg *et al*, 1999; Janzen *et al*, 2003).

The high relapse rates for patients with localised disease following nephrectomy indicate the presence of undetected micrometastasis at the time of surgery (Rodriguez and Sexton, 2006). An enormous challenge is the assessment of the risk of disease recurrence. Although stage, grade, presence of vascular invasion and histological subtype are the currently accepted prognostic indicators, none affords the accuracy required for confident prognostication and therapy selection (Rodriguez and Sexton, 2006; Shuch *et al*, 2006; Park and Eisen, 2007). Additional prognostic indices such as those offered by predictive biomarkers are needed for precise identification of patients at high risk of disease progression and those who will benefit from novel molecular-targeted therapies that have started to emerge for the treatment of RCC (Gore *et al*, 2007; Nelson *et al*, 2007).

Many cellular processes are regulated by AKT including proliferation, mobility, neovascularisation and survival (Samuels and Ericson, 2006). At the plasma membrane, AKT is activated through phosphorylation by phosphoinositide 3-kinase (PI3K) at The308 and Ser473, residues within the AKT kinase domain and the COOH-terminal regulatory tail, respectively (Cheng *et al*, 2005). Stringent control of the PI3K/AKT axis is vital for the prevention of cellular transformation and tumorigenesis and impaired regulation of the PI3K/AKT axis is strongly implicated in oncogenesis (Cheng *et al*, 2005; Yoeli-Lerner and Toker, 2006). Regulation is primarily mediated by phosphatase and tensin (PTEN), a tumour suppressor protein encoded on chromosome 10 (Chow and Baker, 2005). While AKT has many intracellular targets, evidence is accumulating indicating that mammalian target of rapamycin (mTOR) is a crucial downstream effector of AKT either in response to growth factors or through ‘loss-of-function’ mutations in PTEN (Altomare and Testa, 2005).

Mammalian target of rapamycin is an evolutionary conserved multiprotein complex involved in nutrient availability, ribosomal biogenesis and protein synthesis leading to both cell enlargement and proliferation. It was first identified in cells as the pharmacological target of rapamycin, a macrolide antibiotic that inhibits its inherent serine/threonine kinase activity (Hay and Sonenberg, 2004; Tsang *et al*, 2007). Two well-characterised downstream substrates of mTOR are 4E-binding protein 1 (4E-BP1) and the p70 ribosomal S6 kinase (p70S6K). Growth factor mitogen or hormone-stimulated mTOR activation mediates the downstream inhibitory phosphorylation of 4E-BP1 at Ser65 suppressing its ability to bind and inactivate the translation-initiation factor eIF4E. Concomitantly, mTOR mediates the activation of p70S6K, which in turn phosphorylates the 40S ribosomal protein S6 kinase at Ser235 and Ser236, leading to the promotion of mRNA translation. Proteins required for cell-cycle progression such as c-myc and cyclin D1 are activated this way (Averous and Proud, 2006; Mayer and Grummt, 2006).

Previous studies have implicated the AKT/mTOR pathway in a diverse range of cancers including prostate (Kremer *et al*, 2006), lung (Lim *et al*, 2007), pancreas (Asano *et al*, 2005), colon (Nozawa *et al*, 2007) and oesophagus (Hou *et al*, 2007). Tumours that display AKT hyperactivity have shown increased sensitivity to the growth inhibitory effects of rapamycin (Neshat *et al*, 2001). Currently, a number of clinical trials are underway to evaluate the efficacy of various rapamycin derivatives in the treatment of malignancy, either as single or combination therapies (Hutson, 2007; Vogelzang and Sternberg, 2007). Encouraging clinical results have been obtained in RCC to the point that temsirolimus, a rapamycin derivative, has been approved (FDA) in the treatment of advanced RCC (Rini *et al*, 2007).

Caveolin-1 protein was first identified as a substrate for v-src in transformed fibroblasts, it has wide-ranging effects upon signal transduction pathways (Williams and Lisanti, 2004). Numerous *in vitro* studies have demonstrated that caveolin-1 or a peptide domain isolated from it functions as a general ‘molecular brake’ by directly inhibiting the intrinsic kinase activity of numerous growth factor stimulated signalling moieties that include among others the receptor tyrosine kinases (EGFR and PDGFR) and their downstream effectors (Raf, Ras and ERK1/2), nonreceptor kinases (v-src), PKC*α* eNOS and G-protein-coupled receptors (Razani *et al*, 2002; Krajewska and Maslowska, 2004). A number of clinicopathological studies have shown a positive correlation between caveolin-1 overexpression and advanced cancer disease, metatasis and poor prognosis (for critical review see Williams and Lisanti, 2005).

In 2003, we were the first to reveal that overexpression of caveolin-1 in localised RCC predicts poor disease-free survival (Campbell *et al*, 2003). Subsequently, several laboratories have independently examined the expression of caveolin-1 (Horiguchi *et al*, 2004; Joo *et al*, 2004; Phuoc *et al*, 2007) or activated AKT/mTOR/S6 (Lin *et al*, 2006; Pantuck *et al*, 2007; Robb *et al*, 2007) in RCC. However, none has yet examined the expression of these pathways together. We hypothesise that caveolin-1 acts synergistically with the AKT/mTOR pathway in RCC leading to disease progression, and that this molecularly linked signature represents a powerful prognostic determinant for identifying subsets of patients with higher probability of recurrence and metastatic spread. To address this, we examined the coexpression of caveolin-1 and phosphorylated mTOR (pmTOR) pathway components in tumour tissue from 174 RCC patients presenting with localised disease.

## MATERIALS AND METHODS

### Patients and RCC tissue microarray

The patient cohort (174 patients) consisted of a consecutive series of RCCs treated by radical nephrectomy. This series has previously been reported in detail (Thomas *et al*, 2003). None of the patients had received treatment before surgery or had evidence of either lymph node or distant metastatic disease either before or at surgery. Histology reports and slides were available in all cases and reviewed by a pathologist without knowledge of the clinical outcome and assessed for histological type using the Heidelberg classification (Kovacs *et al*, 1997), Fuhrman nuclear grade (Fuhrman *et al*, 1982), the presence or absence of any vascular invasion (either microvascular invasion, renal vein invasion or inferior vena cava invasion) (Griffiths *et al*, 2002) and whether or not there was capsular penetration with cellular invasion of perinephric fat (Thomas *et al*, 2003).

The median age of the patients was 65 (34–88) years; 119 were men and 55 women; 119 of the tumours were conventional (clear cell) carcinomas, 23 were papillary, 5 chromophobe and 27 of the tumours were unclassified by conventional histology. Complete clinical follow-up was carried out as previously described (Griffiths *et al*, 2002; Campbell *et al*, 2003), and the following information extracted from the patients notes date of birth, sex, date of surgery, date last seen, date of death, cause of death and the date on which recurrent or metastatic disease was first identified.

A customised tissue microarray (TMA) was constructed from archived paraffin-embedded tumour samples. A single core, 0.6 mm in diameter, was punched from morphologically representative peripheral regions of each tumour and precisely arrayed into recipient paraffin blocks in a specific orientation. Additional cores were taken from normal renal parenchyma (adjacent to some of the tumours) and human placenta. Sections (4 *μ*m thickness) were cut from the resulting tumour array blocks onto strongly adhesive glass slides (Superfrost Plus™).

### Immunohistochemistry

Immunohistochemical procedures examining for expression of caveolin-1, pAKT (Ser473), pmTOR (Ser2448), pS6 (Ser235/236) and p4E-BP1 (Ser65) were carried out using standard procedures as previously described (Campbell *et al*, 2003). Primary antibodies were obtained from New England Biolabs (Herts, UK). Briefly, TMA sections were deparaffinised, rehydrated and endogenous peroxidase activity within the rehydrated tissue blocked with a solution of 3% hydrogen peroxide in methanol for 10 min at room temperature. Antigen retrieval was carried out by boiling in 10 mM sodium citrate solution (pH 6.0) for 10 min. The TMA slides were cooled and equilibrated in Optimax™ wash buffer then incubated overnight (15 h) at 4°C with primary antibodies for caveolin-1 (rabbit polyclonal, dilution 1 : 10), pAKT (mouse monoclonal, clone 587F11, dilution 1 : 200), pmTOR (rabbit polyclonal, clone 49F9-IHC specific, dilution 1 : 10), p-S6 (rabbit polyclonal, dilution 1 : 100), p4E-BP1 (rabbit polyclonal, dilution 1 : 25) and VHL (rabbit polyclonal, dilution 1 : 10). In all cases, the diluent was 0.6% BSA in Optimax wash buffer. The sections were then washed (4 × 1 min), and the appropriate secondary HRP-conjugated antibody was applied at a dilution of 1 : 100 for 1 h at room temperature with immunoreactivity detected with diaminobenzidene (Sigma, Poole, Dorset, UK) as the chromogenic peroxidase substrate. The slides were counterstained with haematoxylin and mounted.

### Controls and scoring of stained RCC tissue microarray

Evaluation of sections was carried out by consensus over a conference microscope by a pathologist (DFRG) and a research associate (LC) without the knowledge of other pathological and clinical data as previously described (Campbell *et al*, 2003). Expression of each marker was assessed semiquantitatively according to previously validated criteria that accounted for both the intensity of immunostain and percentage of tumour cells involved within each core. Scoring was as follows: (0) no detectable reaction product (deposit) tumour cells; (1) very light diffuse or focal light deposit in tumour cell cytoplasm; (2) light diffuse or moderate focal deposit (but may include very small areas of heavy deposit); and (3) tumour-containing areas of heavy deposit in most or all tumour cells.

Tissue cores from human placenta were used as positive control for pAKT, pmTOR, pS6 and p4E-BP1 immunoreactivity, while staining of peripheral endothelial cells and fat served as positive internal control for caveolin-1. Negative controls run in parallel consisted of TMA sections, where the primary antibody had been omitted or replaced with the appropriate nonimmune serum.

### Data and statistical analyses

Analysis of disease-free survival of patients with tumours showing different scores of staining for each marker was carried out by Kaplan–Meier method using log-rank test, where the first appearance of a metastasis was considered an event. Patients last seen alive without metastasis or who died due to causes other than RCC were considered censored at the date last seen or date of death, respectively. Scores were converted to a binary simple covariate (positive or negative) by thresholding according to the most informative split on the Kaplan–Meier using the log-rank statistical test. For mTOR, AKT and 4E-BP1, a score of 0 was recorded as negative, and a score of 1, 2 or 3 as positive; for SK6 a score of 0 or 1 was negative, and a score of 2 or 3 positive; for caveolin-1 a score or 0, 1 or 2 was negative and a score of 3 positive. The association of the positive biomarkers with recognised tumour prognostic variables (grade, size, vascular invasion, capsular invasion and tumour type) was examined by cross tabulation and the *χ*^2^ test or Fishers exact test as appropriate; for multiple comparisons the Holm–Bonferroni corrections are shown.

To test for synergy between caveolin-1 expression and the activated AKT/mTOR pathway, composite covariates were constructed to indicate dysregulation of both caveolin-1 and activated components of the mTOR pathway. These composite covariates were determined as positive if caveolin-1 was positive and if the selected component of the activated AKT/mTOR pathway was also positive. If either or both were negative the composite covariate was considered negative.

Multivariate survival analysis was carried out by Cox regression using the Enter or Forward Stepwise (Likelihood Ratio) function; with covariates considered as categorical. We had already determined that the most influential covariates predicting the disease-free survival of these patients are Fuhrman grade (grades 1 and 2, and grades 3 and 4 are pooled for analysis), any degree of vascular invasion (histological correlate of stage T2b) and histological invasion of perinephric tissue (the histological correlate of stage T2a). When these covariates are taken into account then tumour size and type had no influence on disease-free survival (Thomas *et al*, 2003). To determine if any of the biomarker covariates had influence on outcome in the multivariate analysis, each (both simple and composite) was added individually in turn as an independent covariate to the Cox regression analysis together with covariates grade, vascular invasion and invasion of perinephric tissues; time to event being the dependent variable. We have previously shown that caveolin-1 upregulation is a good proxy for vascular invasion in multivariate analysis; to determine if this hold true for the composite covariates the Cox regression was repeated omitting the covariate vascular invasion.

The statistical package SPSS 11.5 was used for analysis. All tests were two tailed.

## RESULTS

### Immunohistochemistry for caveolin-1 and activated components of the AKT/mTOR pathway in clinically confined RCC

**Figure 1:**
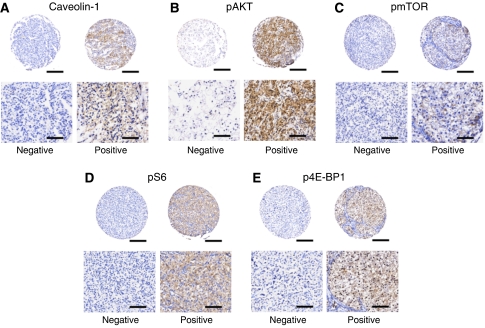
Representative TMA of clinically confined RCC showing the immunohistochemical expression intensity and pattern of (**A**) caveolin-1, (**B**) pAKT, (**C**) pmTOR, (**D**) pS6 and (**E**) p4E-BP1 for tumours that were typically stratified as either ‘Positive’ or ‘Negative’ (see Materials and Methods).

Caveolin-1 staining was comparable to that previously described (Campbell *et al*, 2003) with 28/165 (18%) positive; the distribution was predominately cytoplasmic, although membranous staining was observed in some tumours. Analysis of the activation status of the AKT/mTOR pathway by the detection of phosphorylated forms of each of the individual components showed that 129/166 (78%), 39/97 (40%), 23/107 (21%) and 87/146 (46%) of the RCC patients stained positive for pAKT, pmTOR, pS6 and p4E-BP1, respectively. The staining pattern of all antibodies was essentially similar in nature and consisted of granular and uniform cytoplasmic staining (Figure 1B and E), although some nuclear staining within certain tumours was observed for pAKT and p4E-BP1.

Cross tabulation of caveolin-1, pAKT, pmTOR, pS6 and p4E-BP1 scores with conventional determinants for RCC is shown in Table 1. Increased expression levels of caveolin-1 strongly correlated with tumour grade (*P*=0.019), vascular invasion (*P*=0.001) and tumour size (*P*=0.003). Phosphorylated mTOR correlated with tumour size (*P*=0.023), capsular invasion (*P*=0.015) and with papillary tumour type (*P*=0.006). Of the other AKT/mTOR components evaluated only pS6 demonstrated a significant association with tumour grade (*P*=0.008). Significantly increased expression of pAKT, pmTOR and p4E-BP1 was seen in the papillary RCCs when compared with nonpapillary tumours (Table 1).

### Disease-free survival analysis of caveolin-1 and activated components of the AKT/mTOR pathway in clinically confined RCC

**Figure 2:**
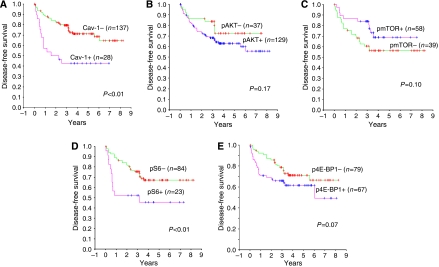
Kaplan–Meier metastasis-free overall survival estimates of RCC patients with clinically confined disease stratified by ‘Positive’ *vs* ‘Negative’ expression of (**A**) caveolin-1, (**B**) pAKT, (**C**) pmTOR, (**D**) pS6 and (**E**) p4E-BP1.

Univariate survival analysis of our series showed that those patients having caveolin-1-positive tumours had significantly worse prognosis with mean disease-free survival of 3.44 years compared to 6.20 years in those with negative caveolin-1 tumours (*P*=0.0005); a finding consistent with our previous investigation (Campbell *et al*, 2003). Kaplan–Meier plots revealed that the expression of cytoplasmic pAKT, pmTOR and p4E-BP1 did not significantly influence disease-free survival. Cumulative survival curves for pAKT, pmTOR and p4E-BP1 are illustrated in Figure 2 and mean disease-free survival values shown in Table 2. In contrast, pS6 was the only component of the activated mTOR pathway that significantly predicted relapse (Figure 2) where the mean disease-free survival in patients whose primary tumours expressed pS6 was 3.77 *vs* 6.11 in those patients whose tumours did not (*P*=0.0096).

### Disease-free survival analysis of combined caveolin-1 and activated components of the AKT/mTOR pathway

**Figure 3:**
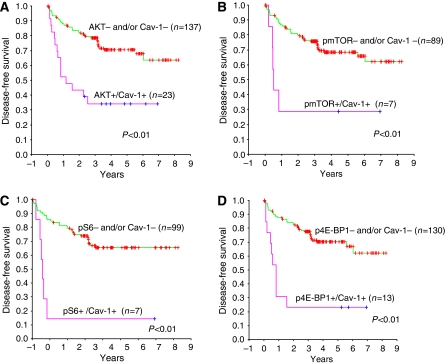
Kaplan–Meier metastasis-free overall survival estimates of RCC patients with clinically confined disease according coexpression of caveolin-1 with (**A**) pAKT, (**B**) pmTOR, (**C**) pS6 and (**D**) p4E-BP1. Patients who coexpressed caveolin-1 and activated components of the AKT/mTOR pathway had significantly worse prognosis compared with patients whose tumours were negative for either biomarker and/or had single biomarker-positive expression.

We next evaluated by Kaplan–Meier analysis the ability of the combined expression of caveolin-1 and each of the other individual downstream-activated components of the AKT/mTOR pathway to predict relapse using the composite covariates previously described. Within the combined caveolin-1/pAKT data set (*n*=160), 23/26 (88%) of the caveolin-1-positive tumours also coexpressed pAKT, while within the combined data sets of caveolin-1/pmTOR (*n*=96), caveolin-1/pS6 (*n*=106) and caveolin-1/p4E-BP1 (*n*=143) the number of caveolin-1-positive tumours that coexpressed the selected individual AKT/mTOR pathway marker was 43, 35 and 52%, respectively (Figure 3).

Patients who had tumours that were positive for both caveolin-1 and pAKT had significantly worse prognosis than patients who had tumours that expressed neither biomarker or either caveolin-1 or pAKT alone, with a mean disease-free survival of 2.95 *vs* 6.14 years (*P*<0.0001). The time to relapse was also substantially shortened for patients whose tumours coexpressed caveolin-1 and pS6, with a survival of 1.45 years compared with 5.96 years (*P*<0.0001). Likewise, the mean disease-free survival for combined caveolin-1 and p4E-BP1 expression was 2.07 *vs* 6.09 years (*P*<0.0001). Coexpression of caveolin-1 with activated pmTOR also significantly reduced the time to disease recurrence with a mean survival of 3.17 *vs* 6.28 years *(P*<0.0001). Collectively, these data demonstrate that patients who had tumours that coexpressed caveolin-1 and an activated AKT/mTOR pathway have significantly shorter recurrence-free survival than those patients who had tumours that expressed caveolin-1 alone or activated AKT/mTOR components alone (Figure 3 and Table 2B).

### Correlation of combined caveolin-1 expression and activated AKT/mTOR pathway components with established RCC clinical/pathological covariates using the composite covariates

To address the association of caveolin-1 expression and a coexisting dysregulated AKT/mTOR pathway with clinicopathological covariates, a composite covariate of caveolin-1/AKT was used. We focused on the biomarker pAKT as an indicator of AKT/mTOR dysregulation as it is generally considered to be the critical upstream effector of the downstream mTOR pathway and represents the central intermediate in our hypothesis that caveolin-1 sustains AKT activation leading to potentiation of the mTOR. Further, neither AKT nor caveolin-1 alone is informative covariates on Cox regression analysis. The caveolin-1/pAKT composite covariate strongly correlated with tumour grade (*P*=0.044), tumour size (*P*=0.008) and vascular invasion (*P*=0.001) (Table 3).

### Cox regression multivariate analysis method

Using multivariate Cox proportional hazard regression models, we evaluated whether the coexpression of caveolin-1 and pAKT could be of prognostic value in the assessment of primary renal tumours. Covariates included in the model were those that have previously shown to be influential, that is, tumour grade, capsular invasion and vascular invasion (Table 4A). The analysis revealed that the composite covariate of ‘caveolin-1 and pAKT’ was a significant influential predictor of shortened disease-free survival with a hazard ratio (HR) of 2.13 (*P*<0.02), whereas the simple covariates caveolin-1 and pAKT alone were not. Of note, this predictive value was higher than that of the powerful and robust prognostic indicator vascular invasion.

We next repeated the computation with vascular invasion omitted to evaluate the prognostic significance of the ‘caveolin-1/pAKT’ covariate for situations where it would not be possible to examine the presence of vascular invasion. In this model, the combined positive expression of caveolin-1 and pAKT had an enhanced HR (2.26; *P*=0.009) than when all other prognostic indices including vascular invasion are incorporated (Table 4B).

Further Cox regression analysis to assess the potential of combining caveolin-1 expression with other activated components of the AKT/mTOR pathway is shown in Table 5. Despite the limited number of valid cases in these data sets, the composite covariates of both caveolin-1/p4E-BP1 and caveolin-1/S6 were both powerful and significant predictors of disease reoccurrence with HR scores of 3.64 (*P*=0.001) and 4.69 (*P*=0.003), respectively.

## DISCUSSION

We have previously shown that increased caveolin-1 serves as a statistically significant indicator of subsequent metastatic disease and poor clinical outcome in patients who initially presented with localised RCC (Campbell *et al*, 2003). Here, in an extended patient cohort (174 patients), we show that caveolin-1 expression strongly correlates with tumour size, tumour grade and the presence of vascular invasion. Further, we show that increased caveolin-1 is associated with reduced disease-free survival, although it is not an influential covariate in multivariate analysis. Importantly, we go on to demonstrate that increased expression of caveolin-1 when combined with an increase in the expression of activated component(s) of the AKT/mTOR pathway represents a more powerful prognostic determinant than either caveolin-1 alone or activated AKT/mTOR component(s) alone, and is highly significant in multivariate analysis.

Several reports have recently examined the expression profiles of various components of the activated AKT/mTOR pathway in RCC (Lin *et al*, 2006; Pantuck *et al*, 2007; Robb *et al*, 2007). However, the heterogeneous nature of their patient groups, which include patients with metastatic disease at presentation, limits the conclusions that can be drawn. In this current work in clinically confined RCC where the patients had received no previous treatment, we found increased pmTOR to demonstrate an association with tumour size and capsular invasion, suggesting that activated mTOR is directly linked to the proliferative capacity of cells within the localised tumour and with tumour invasion into the immediate surrounding tissue such as the renal capsule. In contrast pAKT expression, or for that matter p4E-BP1, was not associated with any histopathological covariates. Only pS6 from the AKT/mTOR pathway predicted a reduced disease-free survival, although when cross tabulated with all other known prognostic determinants pS6 correlated with tumour grade only.

Caveolin-1 has been recognised to potentiate AKT activity in a variety of model systems. We examined the survival analysis within our RCC cohort using caveolin-1 combined as a composite covariate with pAKT (or caveolin-1 combined individually with other downstream mTOR components). The analysis revealed that when caveolin-1 is coexpressed with either pAKT, pmTOR, pS6 or p4E-BP1 within the primary tumour, time to relapse was significantly reduced compared with when either of the individual variables were expressed alone. The critical upstream effector of the mTOR pathway, pAKT, as a composite parameter with caveolin-1, was selected for further examination against established clinicopathological parameters. Despite the frequent overexpression of activated AKT in primary tumours, its expression alone in RCC had no significant correlation with histopathological parameters. However, the composite covariate of ‘caveolin-1 and activated AKT’ correlated with tumour size, grade and the presence of vascular invasion in an independent manner and to a statistically greater extent than when either variable, caveolin-1 or pAKT was analysed alone. We interpret from this that patients with localised RCC whose tumours are positive for both caveolin-1 and pAKT are likely to have more aggressive and vasoinvasive disease. Further, this demonstrates that the composite covariate of caveolin-1 and activated AKT serves as a linked molecular signature to identify localised tumours that have an increased propensity to metastasise and provides substantial support for a hypothesis that caveolin-1 acts synergistically with the AKT/mTOR pathway in driving disease progression in RCC.

In our previous work (Campbell *et al*, 2003), we found that caveolin-1 can be substituted for vascular invasion in a hazard model for time to recurrence (but not for grade or capsular invasion), suggesting that its expression is linked to vascular proliferation. Although the specific mechanism by which caveolin-1 facilitates cancer progression is still unclear, the strong correlation with vascular invasion suggests that caveolin-1 contributes to angiogenesis and metastasis. Vascular invasion is considered to be one of the most important and robust pathological variables for RCC evaluation (Griffiths *et al*, 2002; Gonçalves *et al*, 2004; Dall'Oglio *et al*, 2007). Therefore, we constructed a multivariate Cox regression Hazard model to determine if coexpression of caveolin-1 and pAKT could be a superior prognosticator in a preoperative assessment scheme. In such an analysis, we found that the composite covariate ‘caveolin-1/pAKT’ can be substituted for vascular invasion and indeed provides for as good a model as vascular invasion in predicting disease recurrence. This covariate would serve as a readily determined biomarker for the assessment of tumour recurrence in clinically confined RCC when histopathological variables cannot be that easily assessed, for example, in limited biopsy material or in tumours morcellated *in situ* (Rabban *et al*, 2001).

The clinical course of RCC patients that present with localised disease is difficult to predict, even within subgroups with seemingly similar histopathological characteristics. While tumour biology clearly mirrors that of its histopathological features, it is suggested that the molecular profile of the tumour more accurately reflects the biological behaviour of the tumour (Kim *et al*, 2005). Therefore, individual biomarkers and linked molecular signatures such as those identified in our present study can clearly supplement standard clinical information to provide enhanced prognostication of RCC patients. To this end, two recent innovative studies have reported the development of a prognostic model that incorporates a panel of malignancy-associated biomarkers (which includes p53, CA9, Ki67, vimentin, CA12, PTEN and gelosin) with standard clinical parameters and where the resultant ‘clinical/molecular marker’ model, better predicts survival of patients with RCC (Kim *et al*, 2004, 2005). Specifically, when ranked alongside several conventional RCC staging systems such as Fuhrman grade, TNM and ECOG-Performance Status (PS), the integrated ‘clinical/molecular marker’ prognostic model demonstrated superior prognostic value than either and/or that afforded by the panel of biomarkers alone. Of note, the ECOG-PS of our patient cohort was not available, and since this represents an important model for predicting prognosis of RCC patients (Kim *et al*, 2004; Phuoc *et al*, 2007), its omission should be acknowledged as one limitation to our current Cox proportional hazard regression modelling. Nevertheless, the composite covariates of caveolin-1 and activated AKT/mTOR components serve as linked molecular signatures that clearly identify localised tumours, which have high invasive capacity and an increased metastatic potential. Therefore, it is envisaged that their incorporation within similar combined staging systems for RCC as described above will further improve the predictive power of such models.

Interestingly, a subset analysis of our RCC cohort revealed that the expression of caveolin-1 was invariably absent or minimal within papillary carcinomas; an observation in agreement with Tamaskar *et al* (2007). However, high expression of pAKT, pmTOR, pS6 and p4E-BP1 was evident in these papillary tumours. Papillary carcinomas represent a subtype that are usually less vasoinvasive, have reduced metastatic potential and generally have a good prognosis (Mai *et al*, 2001; Cheville *et al*, 2003). Our data therefore suggest that the presence of an activated AKT/mTOR pathway alone is not sufficient to impart an aggressive phenotype but requires caveolin-1, at least in RCC. Consistent with this finding, *in vivo* studies show that caveolin-1 knockout mice have a number of relevant features: (i) when inoculated with the exogenous melanoma cell line B16-F10, the caveolin-1 knockout mice show an impaired angiogenic response with reduced tumour weight, volume, blood vessel infiltration and microvessel density compared with wild-type littermates (Woodman *et al*, 2003); and (ii) when interbred with TRAMP mice that spontaneously develop advanced prostate cancer and metastatic disease, the resultant strain that displays inactivation of one or both caveolin-1 alleles has reduced tumour burden and significant reductions in regional lymph node and distant organ metastasis (Williams *et al*, 2005).

An association between the expression of caveolin-1 and AKT has been previously reported in clinical tumours of the colon (Kim *et al*, 2006). Although the relationship on disease progression was not evaluated, the authors suggested that the presence of caveolin-1 positively affects AKT activation. Support for the clinical relevance of caveolin-1/AKT interaction and its impact on the downstream targets such as mTOR comes from a series of *in vitro* experiments that directly demonstrate that caveolin-1 has the capacity to facilitate AKT signalling in different cell types (Li *et al*, 2003; Sedding *et al*, 2005; Zhang *et al*, 2007). From the perspective of human cancer progression, Li *et al* (2003) showed that caveolin-1 overexpression was able to maintain activated AKT levels in human prostate cancer cells via inhibition of the tumour suppressor enzymes PP1 and PP2A, which dephosphorylate AKT at both Ser473 and The308. Furthermore, the caveolin-1-mediated increases in AKT activity were sufficient to cause the sequential phosphorylation of multiple downstream AKT substrates that included GSK3, FKHR and MDM2 (Li *et al*, 2003). While the regulation of the mTOR pathway by AKT was not examined in the latter study, several lines of investigation provide data to indicate that this is highly probable and that analogous mechanisms also occur in RCC given that PP1 has recently shown to have important growth inhibitory properties in RCC-derived cell lines (Fujimoto *et al*, 2006; Hagiwara *et al*, 2006).

**Figure 4:**
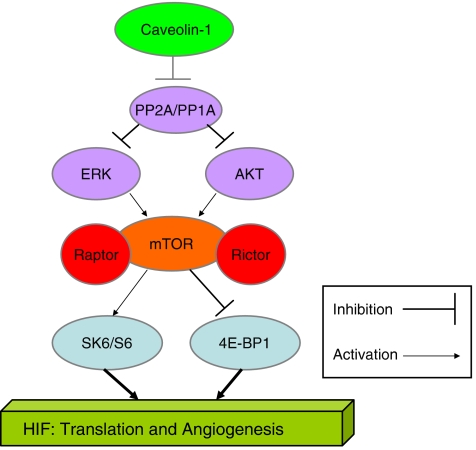
Schematic of the putative caveolin-1/AKT/mTOR axis in RCC.

We propose that caveolin-1 may serve as a ‘gatekeeper’ for activation of the hypoxia-inducible factor (HIF) pathway a downstream effector molecule of mTOR that accumulates in RCC in response to the loss of function of VHL and promotes angiogenesis, vascular invasion and chemoresistance (Patel *et al*, 2006). Support for this notion is in part provided by other clinical studies that show that the overexpression of caveolin-1 is correlated with increased microvessel density within tumours not only of the kidney (Joo *et al*, 2004) but also the prostate (Yang *et al*, 2007). Therefore, we present a hypothetical model (adapted from Li *et al*, 2003) for the coordinated role of caveolin-1 and the AKT/mTOR pathway in RCC disease progression. In this, caveolin-1 sustains the activation of the mTOR pathway leading to HIF-mediated angiogenesis via upstream inhibition of a protein phosphatase that in turn causes loss of repression of both AKT and ERK1/2 (see schematic Figure 4). Although we have not yet examined the correlation between activated ERK1/2 and caveolin-1 in RCC, clearly, the ERK1/2 pathway appears to converge on the mTOR pathway in RCC, given that specific inhibitors of EGFR sensitise RCC cell lines to the effects of rapamycin (Costa *et al*, 2007). That caveolin-1 and mTOR may directly constitute a signal transduction ‘cassette’ is supported by analysis of the published peptide sequences of raptor (GenBank accession no. NP065812) and rictor (GenBank accession no. AAH51729.1), two scaffold proteins essential for mTOR function that serve to bring mTOR substrates into close proximity to its catalytic domain. We are able to identify that both raptor and rictor contain a recognised consensus caveolin-1-binding motif (ΦxxxxΦxxΦ, where Φ is an aromatic residue and x is any amino acid) (Couet *et al*, 1997). Within raptor, the main accessory molecule of the rapamycin-sensitive mTOR pathway can be identified by the motif L-xxxx-F-xx-F located between amino acids 201–209. This, although not a bone fide caveolin-binding motif, has been shown to be critical in mediating a functional association between caveolin-1 and G-protein-coupled receptor kinases (Carman *et al*, 1999). The corresponding caveolin-binding domain in rictor, the central scaffold protein of the rapamycin-insensitive pathway is present between amino acids 889–897(**F**QDIP**Y**SD**W**).

In conclusion, the coexpression of caveolin-1 and activated components of the AKT/mTOR pathway represents a ‘linked molecular signature’ that identifies patients with localised RCC that are at high risk of developing metastatic disease that warrants greater postoperative surveillance. Evaluation of the expression status of both caveolin-1 and mTOR pathway components in these tumours may help predict tumour response to novel pathway-specific therapies, hence allowing appropriate selection of treatment for individual patients. In addition, these current results also further underline the importance of caveolin-1 in RCC progression and its validity as a future therapeutic target for advanced RCC.

## Figures and Tables

**Table 1:** Relationship of caveolin-1, pAKT, pmTOR, pS6 and p4E-BP1 expression with conventional clinicopathological parameters in clinically confined RCC

	**Caveolin-1 (*n*=165)**		**pAKT (*n*=166)**		**pmTOR (*n*=97)**		**pS6 (*n*=107)**		**p4E-BP1 (*n*=146)**	
	**−ve**	**+ve**	**−ve**	**+ve**	**−ve**	**+ve**	**−ve**	**+ve**	**−ve**	**+ve**
Grades 1 and 2	102	14	29	89	40	30	64	11	60	45
Grades 3and 4	35	14	8	40	18	9	20	12	19	22
		*P*=0.01		*P*=0.535		*P*=0.39		*P*=0.008		*P*=0.23
										
*Tumour size*										
<7 cm	72	6	20	61	25	26	42	11	36	33
>7 cm	65	22	17	68	33	13	42	12	43	34
		*P*=0.003		*P*=0.468		*P*=0.023		*P*=0.853		*P*=0.657
										
*Vascular invasion*										
(−ve)	90	9	24	79	31	26	52	9	45	44
(+ve)	47	19	13	50	27	13	32	14	34	23
		*P*=0.001		*P*=0.689		*P*=0.195		*P*=0.051		*P*=0.283
										
*Capsular invasion*										
(−ve)	118	20	31	109	42	36	71	16	64	56
(+ve)	19	8	6	20	16	3	13	7	15	11
		*P*=0.06		*P*=0.916		*P*=0.015		*P*=0.103		*P*=0.686
										
Nonpapillary	116	27	37	106	54	28	76	16	77	49
Papillary	21	1	0	23	4	11	8	7	2	18
		*P*=0.096	*P*=0.005^*^	(*P*=0.095)	*P*=0.006^*^	(*P*=0.10)	*P*=0.94		*P*<0.000^*^	(*P*=0.01)

pAKT=phosphorylated AKT; 4E-BP1=4E-binding protein 1; p4E-BP1=phosphorylated 4E-binding protein 1; RCC=renal cell carcinoma; mTOR=mammalian target of rapamycin; pmTOR=phosphorylated mammalian target of rapamycin.

Unequal numbers are due to occasional loss of tumour in array spots on some slides, and, for pmTOR and pS6, failure to achieve immunocytochemical reactivity with tissue derived from for one of the two laboratories. Associations considered significant at *P*<=0.10 after correction for multiple comparisons are shown with ^*^ and with appropriate corrected *P*-values in parentheses (caveolin results are a confirmation of previous findings – correction for multiple comparisons is not appropriate).

**Table 2:** Mean metastasis-free survival of patients with clinically localised renal cell carcinoma

**(A) Prognostic indices/univariate model**	**Mean survival (years)**	**95% CI**	***P*-value**
Caveolin-1 positive (28)	3.44	2.31–4.58	0.0005^*^
Caveolin-1 negative (137)	6.20	5.64–6.76	
AKT positive (37)	5.52	4.89–6.15	0.1779
AKT negative (129)	5.94	5.04–6.85	
mTOR positive (58)	5.82	2.02–7.29	0.103
mTOR negative (39)	5.21	4.29–6.14	
S6 positive (23)	3.77	1.27–4.71	0.0096^*^
S6 negative (84)	6.11	5.41–6.81	
4E-BP1 positive (67)	5.09	4.15–6.03	0.077
4E-BP1 negative (79)	5.21	4.29–6.14	
			
**(B) Prognostic indices/composite covariate model**	**Mean survival (years)**	**95% CI**	***P*-value**
Caveolin-1/AKT positive (23)	2.95	1.74–4.16	<0.0001^*^
Caveolin-1/AKT negative (137)	6.14	5.57–6.70	
Caveolin-1/mTOR positive (7)	3.17	2.01–4.33	<0.0001^*^
Caveolin-1/mTOR negative (89)	6.28	5.74–6.83	
Caveolin-1/S6 positive (7)	1.45	0.00–3.12	<0.0001^*^
Caveolin-1/S6 negative (99)	5.96	5.57–6.7	
Caveolin-1/4E-BP1 positive (13)	2.07	0.6–3.53	<0.0001^*^
Caveolin-1/4E-BP1 negative (130)	6.09	5.29–6.62	

4E-BP1=4E-binding protein 1; CI=confidence interval; mTOR=mammalian target of rapamycin.

^*^Denotes significance *P*<0.05.

**Table 3:** Correlation of combined caveolin-1 and pAKT (caveolin-1/AKT covariate) positivity with clinical and pathological parameters in patients with clinically localised renal cell carcinoma

**Clinicopathological parameter**	**Caveolin-1 and AKT negative (*n*=137)**	**Caveolin-1 and AKT positive (*n*=23)**	***P*-value**
Tumour grades 1 and 2	100	12	0.044^*^
Tumour grades 3 and 4	37	11	(0.088)
Tumour size <7 cm	71	5	0.008^*^
Tumour size >7 cm	66	18	(0.032)
Vascular invasion (−ve)	90	7	0.001^*^
Vascular invasion (+ve)	47	16	(0.004)
Capsular invasion (−ve)	117	17	0.167
Capsular invasion (+ve)	20	6	

Associations considered significant at *P*<=0.10 after correction for multiple comparisons are shown with ^*^, with appropriate corrected *P*-values in parentheses.

^*^Denotes significance *P*<0.05.

**Table 4:** Multivariate Cox regression hazards model for time to recurrence

**(A) Prognostic indices/model including vascular invasion {enter function} (*n*)**	**HR**	**95% CI**	***P*-value**
Grades 1 and 2 (112)	1		
Grades 3 and 4 (48)	2.94	1.62–5.36	<0.01^*^
No capsular invasion present (134)	1		
Capsular invasion present (26)	4.22	2.26–7.90	<0.01^*^
No vascular invasion present (97)	1		
Vascular invasion present (63)	1.74	0.88–3.46	0.113
Caveolin/AKT covariate negative (137)	1		
Caveolin/AKT covariate positive (23)	2.13	1.15–3.92	<0.02^*^
			
**(B) Prognostic indices/model excluding vascular invasion {forward conditional} (*n*)**	**HR**	**95% CI**	***P*-value**
Grades 1 and 2 (112)	1		
Grades 3 and 4 (48)	3.58	2.05–6.24	<0.001^*^
No capsular invasion present (134)	1		
Capsular invasion present (26)	5.17	2.88–9.29	<0.001^*^
Caveolin/AKT covariate negative (137)	1		
Caveolin/AKT covariate positive (23)	2.26	1.23–4.16	0.009^*^

CI=confidence interval; HR=hazard ratio.

^*^Denotes significance *P*<0.05.

**Table 5:** Hazard ratio for time to recurrence of each composite covariate as calculated by separate multivariate Cox proportional hazards method using the forward conditional function (including tumour grade, vascular invasion and presence of caveolin/AKT/mTOR pathway covariate)

**Prognostic indices/covariate model (*n*)**	**HR**	**95% CI**	***P*-value**
Caveolin-1/mTOR (7)	2.61	0.99–6.82	0.051
Caveolin-1/4E-BP1 (13)	3.64	1.75–7.54	0.001^*^
Caveolin-1/S6 (7)	4.69	1.17–12.8	0.003^*^

4E-BP1=4E-binding protein 1; CI=confidence interval; mTOR=mammalian target of rapamycin.

In all grades and capsular invasions HR remain significant.

^*^Denotes significance *P*<0.05.
